# Ensuring Purpose Limitation in Large-Scale Infrastructures with Provenance-Enabled Access Control

**DOI:** 10.3390/s21093041

**Published:** 2021-04-26

**Authors:** Shizra Sultan, Christian D. Jensen

**Affiliations:** Department of Mathematics and Computer Science, Technical University of Denmark, Anker Engelunds Vej 1, Building 101A, 2800 Kongens Lyngby, Denmark; cdje@dtu.dk

**Keywords:** privacy, compliance, data protection, provenance, purpose limitation, secondary use

## Abstract

The amount of data generated in today’s world has a fair share of personal information about individuals that helps data owners and data processors in providing them with personalized services. Different legal and regulatory obligations apply to all data owners collecting personal information, specifying they use it only for the agreed-upon purposes and in a transparent way to preserve privacy. However, it is difficult to achieve this in large-scale and distributed infrastructures as data is continuously changing its form, such as through aggregation with other sources or the generation of new transformed resources, resulting often in the loss or misinterpretation of the *collection purpose*. In order to preserve the authorized *collection purposes*, we propose data is added as a part of immutable and append-only resource metadata (provenance), to be retrieved by an access control mechanism when required for data-usage verification. This not only ensures purpose limitation in large-scale infrastructures but also provides transparency for individuals and auditing authorities to track how personal information is used.

## 1. Introduction

During the past decade, the invasive presence of IoT, social media websites, and smart-city services has emphasized the importance and usefulness of personal information. Personal information refers to any physical, physiological, biometric, or digital piece of information that can link a natural entity to a unique identity [1]. A large number of data applications and services collect and process personal information about individuals via different sources to provide them with personalized and informed services. Misuse or misrepresentation of any piece of personal information by a data-collecting entity, or any use of personal information without individuals’ consent or a valid legal base, is a legal violation leading to privacy invasion. To protect personal information, countries around the globe have introduced several data-protection legislations such as the EU General Data Protection Regulation (GDPR), the California Consumer Privacy Act (CCPA), etc. [2,3]. These legislations provide a legal framework for data owners or data controllers (DCs) regarding the use and protection of the personal information of individuals or data subjects (DSs), declaring data reuse for any purpose other than the agreed-upon as a violation, i.e., purpose limitation. Furthermore, it also underlines the rights of DSs over their personal information, such as their right to be informed about how their information is being used, and a right to object if it is not used accordingly. Thus, it is a legal obligation of a DC to ensure purpose limitation by collecting and using personal information only for agreed-upon purposes. Purpose can here be distinguished into two categories. First, the *collection purpose,* referring to the terms of an agreement between the DC and the DS about resource usage, i.e., why the data (specifically personal information) is being collected, how much of it will be stored and used, etc. Second, the *access purpose*, referring to the terms of an agreement between the DC and the data-users or data processors (DPs) describing how can DPs use data and under what limitations [4]. In order to ensure purpose limitation, an *access purpose* of a DP should comply with the *collection purpose* of the resource.

Large-scale integrated infrastructures store petabytes of data or resources contributed by multiple DCs for different *collection purposes*, and DPs then request such data for different authorized *access purposes* [5]. The data in shared infrastructure is altered, transformed, and aggregated with data from other DCs several times to generate new insights and retrieve information relevant to the DP’s requirements. This raises certain concerns when it comes to ensuring purpose limitations in the distributed and information-sharing environment. First, due to frequent changes in the structure and content of the data, often the *collection purposes* of the data are lost, misinterpreted, or not preserved appropriately, leaving a gap for biased interpretation [6]. Second, DCs may not have control over all the data/resource transformations in a distributed or shared environment, and often it is hard to constantly authorize *access purposes* for a DP requesting data in different forms and accommodate their emerging requirements. For instance, a DP may request an aggregation of different resources, but it is possible that its existing *access purpose* will not comply with the *collection purposes* of the newly aggregated resource, thus denying rightful access to that data [7]. Third, often the DC to which the requested resource belongs does not regulate all the requester DPs registered in large-scale infrastructure. In cases such as this, an individual DC’s *access purposes* may be designed or defined differently by another DC that may authorize them to request resources, thus allowing them access to data that might be incompatible in terms of *collection purposes*. Thus, if both the *collection purpose* and the *access purpose* are inconsistent, and do not follow similar formats or characteristics, they are often incompatible with each other, and based on lenient or stricter policies this may either allow secondary use or prohibit authorized use. To conclude, large-scale integrated infrastructures often fail to ensure purpose limitation due to unsuccessful verification between the different *collection purposes* of resources and DP’s *access purpose*, because of inconsistent definitions by different DCs. Moreover, due to frequent data transformations and aggregations, often *collection purposes* are misinterpreted or miscommunicated leading to data-protection guideline violations, resulting in secondary use and privacy invasion. Current state-of-the-art solutions designed for large-scale infrastructures lack a comprehensive solution that addresses the mentioned problems simultaneously.

In order to address the above-mentioned concerns, we present two arguments; first, that the representation of purpose (both collection and *access purpose*) should follow some standard format so they can be verified against each other as per the requirements of the applied data protection guidelines to ensure purpose limitation, especially in distributed infrastructures with diverse DCs. Second, a key requirement in purpose limitation is purpose integrity, or more specifically *collection purpose* integrity, and thus this should be preserved. Purpose limitation ensures that resource usage is strictly governed by its *collection purpose*; however, in the case of frequent transformations and aggregations, it is often not well-preserved, thus defeating purpose limitation. Thus, it is important to preserve the integrity of the *collection purpose*, i.e., ensure that this is exactly the same as agreed upon between the DC and DS, and second, that it is readily available to DPs in its conserved state whenever the resource is requested by an authorized DP.

In this paper, we propose a framework for representing, storing, and aggregating the *collection purpose* of a resource as per commonly observed data-protection guidelines, and demonstrate how an *access purpose* can be verified against this to ensure purpose limitation. Moreover, we further propose to add the said *collection purpose* as an immutable resource property (provenance), to preserve its integrity through different resource transformations. The provenance is a resource (metadata) property that catalogs different activities that are performed on a resource along with its lineage and are often immutable and append-only. The provenance will initially record the *collection purpose* along with resource origin, and then with every transformation or aggregation, the *collection purpose* will be preserved and appended (updated) if required, ensuring purpose integrity. Furthermore, as the *collection purpose* will itself be a resource property, it will be readily available with the resource in its preserved state, when requested by a DP, leaving less to no room for misinterpretation. Therefore, our proposed framework ensures compliance with different data protection legislation by ensuring purpose limitation and preserving purpose integrity that not only limits secondary usage but also builds up trust among DCs, DPs, and DSs as regards resource usage transparency.

To illustrate the above-mentioned issues in a large-scale and distributed infrastructure, we use a smart-city traffic management system as a motivational example throughout this paper. This example is presented in Section 2. Section 3 discusses provenance in detail and proposes a framework to record and preserve *collection purposes* in data aggregations by using the motivation example discussed in Section 2. Section 4 analyzes how provenance enables an access control mechanism that implements purpose limitation and restricts secondary use in large-scale infrastructures, followed by Section 5 which highlights a related state-of-the-artwork, with Section 6 presenting a conclusion.

## 2. Motivation Example

**Figure 1:**
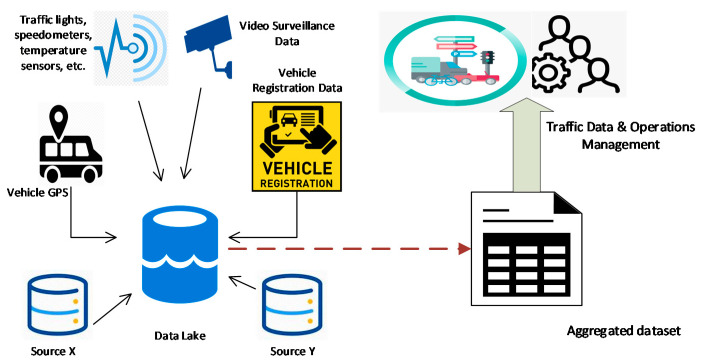
The SC-TMS integrates data from multiple DCs.

Here, we will discuss a smart-city traffic management system (SC-TMS) as a motivation example that imitates a large-scale distributed infrastructure. The SC-TMS aggregates data from various public DCs in heterogeneous formats collected for various *collection purposes*, supported by a valid legal base. A legal base establishes legal grounds for personal data processing activities and is a must requirement by various data-protection legislation. Various resources contribute to the SC-TMS with different *collection purposes*, for instance, video surveillance recordings monitor traffic operations, time-series location data from public transportation manages routes and traffic congestion, vehicle registration data handles traffic violations and missing vehicles, etc., as shown in Table 1. [4]. Similar systems are being used in all the major cities of the world to regulate traffic operations with real-time traffic patterns to manage congestion and traffic issues, helping city administrations upgrade the transportation infrastructure in a manner which coincides with citizens’ requirements. Many smart-city authorized DPs such as traffic officers, traffic-law enforcement systems, congestion handlers, route-planners, infrastructure-planning departments, etc., can request the SC-TMS to combine data from different DCs in order to generate results that fulfill their requirements or serve their *access purposes*, as shown in Figure 1 [5].

Different resources have different information to offer, and many of these resources contain personal information in different forms, which when analyzed together can reveal valuable and enriched personal information [7]. Thus, because personal information is involved, the SC-TMS must preserve the privacy of individuals and use their data only for agreed-upon *collection purposes*. However, most of the SC-TMS data is collected under the legal base “public interest” or “legal obligation”, and as these DCs (more or less) belong to public authorities, their *collection purposes* are also related to public infrastructures and operations. However, it does not mean that all public authority DPs have unlimited access to this data, and their authorized requirements or *access purposes* should always be a subset of the requested resource’s *collection purpose*. For example, a route-planner DP has an *access purpose* to use GPS information of different vehicles in order to determine the optimal route from point A to point B. There is also other information that can be obtained from the same resource with a valid *collection purpose*, such as past trip durations, frequently visited locations, vehicle-parking logs, vehicle/driver current location, etc. Let us assume in this case that the DP (route planner) is not authorized to access or use the available personal information for any purpose other than route planning. This example shows that DPs with authorized access to the SC-TMS data are often exposed to more information than they require and thus have the potential to use the available information for purposes other than those agreed upon, i.e., secondary use. In order to avoid this, ideally and legally, DPs should only be able to use the authorized resources or information as per their *access purposes* that corroborate with the *collection purpose* of the resource, i.e., purpose limitation in order to preserve privacy. In order to achieve this, it is important that the DC responsible for the resources with personal information should represent the resource’s *collection purpose* in a way, which is comprehensible for the SC-TMS so it can be observed and verified against the DP’s *access purpose*.

On the other hand, due to frequent transformational changes in data, often DPs with authorized *access purposes* are unable to access data due to the lack of required *collection purpose* preservation. For example, DPs who work in the traffic-law enforcement system are allowed to access both the vehicle registration database and the video surveillance system separately in order to access the required data (about traffic violations) relevant to both resources’ *collection purposes*. Let us assume a case in which a DP has requested an aggregation of the above-mentioned resources. Theoretically, the DP should be allowed to access the aggregated resource for its already authorized *access purpose* that is an overlap of the *collection purposes* of both involved resources, and thus this use would not be a privacy violation or secondary use. However, the SC-TMS, in order to limit secondary use, does not let the DP access the aggregated resource, as either the aggregated resource has undefined *collection purpose* or requires a DC to authorize a new *collection purpose* every time their contributed resource is transformed, even when the access is being requested by an already authorized DP. To avoid the need for continuous authorization by DCs or the declination of a rightful request of a DP, it is necessary to preserve the *collection purpose* of the resource in a way that ensures it is readily available when the resource is requested, even if it is transformed or aggregated.

In city-scale infrastructure with cross aggregations between different data sources managed by different DCs it is often hard for any DC to be aware of the current and future *access purposes* of thousands of DPs and manage their authorizations continuously. Moreover, data changes into multiple forms in distributed systems, and often the *collection purpose* is overlooked during different transformations and aggregations. Therefore, if the resource preserves the integrity of that *collection purpose* as part of its indisputable metadata (provenance), it can assist the SC-TMS in establishing that the *access purpose* complies with the *collection purpose* and thus ensures purpose limitation. Moreover, in the case of different data aggregations, where the same DC does not manage resources or *collection purposes* of the involved resources, DPs can use the preserved *collection purposes* derived from the provenance for relevant or compatible *access purposes* without violating purpose limitation. The next section will discuss how provenance is created and how it will record and preserve *collection purpose* as a provenance property.

## 3. Provenance and Collection Purposes

To store and organize different types of data/resources contributed by different DCs, large-scale distributed infrastructures usually have some primitive metadata schema that identifies the basic structure or nature of the data content without going into granular details. Often these schemas also store information about data or resource lineage, i.e., by tracking different activities or processes that data goes through from its origin to consumption by different DPs. It also stores information about who collects and owns the data, how long it should be stored for, how different transformations are cataloged, etc., and is often referred to as provenance [8]. Typically, metadata of any resource can be modified at any point during the data lifecycle, though, provenance metadata is often considered immutable and append-only, requiring systems to efficiently manage and preserve it through different transformations [9]. Moreover, when data from different DCs is aggregated, it is anticipated that their provenances will also be integrated or stitched in a logical manner. Hence, provenance is a useful way to record and catalog different changes in a data lifecycle, especially in large-scale shared infrastructures where a DC does not have complete control over data transformations.

**Figure 2:**
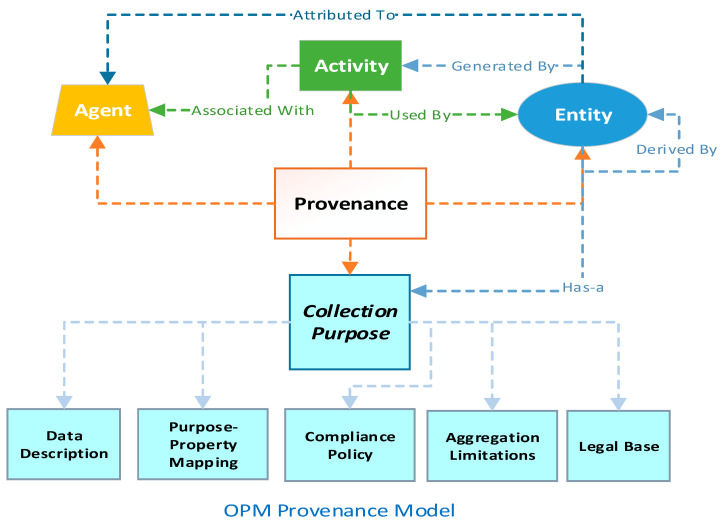
Provenance model.

When a resource is created or inserted into a system, its provenance metadata is created too, and with every action/activity performed over a resource an entry is created and stored against it. The provenance is built on three main concepts: agent, activity, and entity, as shown in Figure 2, while in an earlier paper we established that *collection purposes* can be the fourth key concept recorded in provenance if an entity contains personal information [10]. The activity is an action or any type of processing activity that creates, modifies, or deletes an entity. An entity is a data resource or object in any format that can be processed. Thirdly, the agent here is an actor (DC or DP), that has the authority to perform an activity over an entity. Lastly, the *collection purpose* contains information on how an agent can use the personal information present in an entity and is defined by five characteristics, as shown in Figure 2. The motivation behind choosing these five characteristics to describe the *collection purpose* is to show compliance with the current data protection legislation. For instance, a recent and comprehensive data protection legislation, the GDPR, states the notion of purpose and its limitation in the below-mentioned articles or stipulations, as shown in Table 2 [2].

The data minimization article states that any data collected should be relevant and specific for the usage purpose, thus, to achieve this it is important to understand data and its properties so that personal properties can be distinguished, hence the first characteristics of “data description” (identifying resource attributes/identifiers that store personal information). Second, “purpose-property matching” adheres to both data minimization and purpose limitation by binding identified personal properties to specific and explicit functions, so that they cannot be used otherwise (i.e., mapping purpose-functions with the required set of identifiers). Third, “compliance policy” and “aggregation limitations” specify the conditions and limitations upon how the purpose-property matching functions can be used, further describing the conditions that a DP needs to fulfill in order to access specific data properties for an explicit function following an access control article. The last characteristic records the “legal base”, as it is crucial to decide what rights a DS can execute over personal information. Thus, the definition of *collection purpose* is in accordance with the GDPR, and more or less all the recent legislation has the same requirements. These characteristics are discussed in detail in a previous paper [10].

Over the years different provenance schemes have been proposed to describe methods that to show data lineage and derivation [8,9]. These schemes may offer a different view of provenance metadata based on use, i.e., debugging, reproducibility, annotation, security, etc. One of the most successfully used schemes or models to store provenance is the open provenance model (OPM) that stores provenance entries as directed acyclic graph (DAG) nodes to describe the chronological workflow of all the activities that are performed over a resource (entity) by an agent [11]. An entity, agent, activity, and in this case the *collection purpose*, are all labeled as vertices of the DAG, while the edges of the DAG show the relationship among these vertices. Therefore, when an entity is created, the first provenance entry contains information about the resource (entity), its creation (activity), when and who created it (agent), and why the resource has been created (*collection purpose*). Later, entries are created whenever data goes through some transformation or any activity is performed over the entity, and this information becomes part of the provenance DAG or hierarchy.

### 3.1. Provenance Preservation

In large-scale distributed infrastructures, entities from different resources are transformed multiple times to fulfill the requirements of the DC, which may then be shared among multiple DCs, and are available for DPs with diverse *access purposes*. The DS whose personal information is recorded in those entities has given either informed or forced consent (as a legal obligation) that their personal information may only be used for the agreed-upon *collection purpose*. However, many DCs are involved in managing large-infrastructures, and not every DC will be forthcoming to allow another DC to examine how the shared resource or entities are being used, which eventually decreases the trust of a DS over the use of their personal information as per agreed-upon *collection purposes*. We propose to utilize resource provenance to also record its *collection purpose/s*, and that any DP requiring access to a resource must comply with the *collection purpose/s* available at the time of the request. Furthermore, after the request is granted, the provenance will also record the *access purpose* of the agent for the performed activity. As the provenance is considered immutable and append-only, it will be retained through different transformations, providing a way for any DC to trace and review the usage of its entities [12]. This provides both the prevention of secondary use as well as a way to ensure *collection purpose* verification. For the former objective, resource provenance can be used in access control decisions, where the *collection purpose* can be retrieved from the provenance of the resource and can be compared with the *access purpose* of the requester, and if compliant then access can be granted. For the latter objective, a DC at any point of the resource’s lifecycle can confirm by reviewing the provenance metadata whether the entities’ *collection purpose* was comparable to the *access purpose* of the agent.

### 3.2. Provenance in Aggregated Resources

It is a common occurrence for a DP in shared infrastructures to request an aggregation of different resources to generate a new entity that holds information from all the parent resources [13]. Technically, these resources are independent of each other and thus have independent or separate provenances. Once the resources are aggregated, then their provenances are also (preferably) aggregated or stitched together. Newly aggregated or transformed data that contains personal information requires a valid or declared *collection purpose* to be requested or accessed by any agent (DP) with an authorized *access purpose*. Therefore, if different resources and their provenances are stitched, their *collection purposes* should also be (ideally) aggregated and preserved.

**Figure 3:**
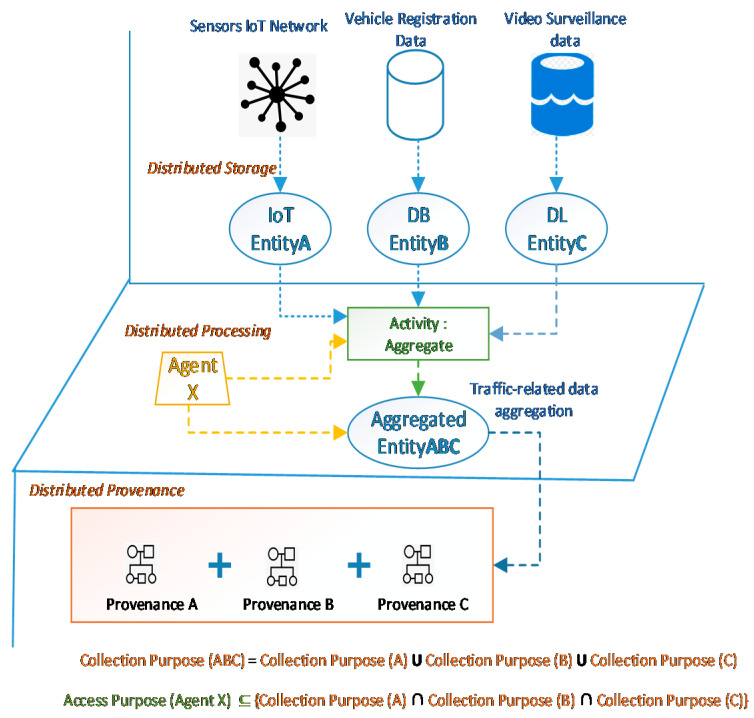
Provenance preservation in aggregated resources.

Referring to the motivation example presented in Section 2, here we use it as an example to show how provenance is retained, as shown in Figure 3. An authorized DP (traffic law enforcement system TLE) requests an aggregation of three authorized resources, i.e., video surveillance data (entity A), vehicle registration data (entity B), and vehicle sensors data (entity C). In order to prevent secondary use and a potential breach of confidence, any DP (or in this example an agent TLE) requesting the aggregated resource must have authorization or an *access purpose* that is compatible with the *collection purposes* of all the parent resources implicitly, unless otherwise explicitly specified for the new resource. The explicit *collection purposes* are designed and followed when the aggregation or transformation is expected at some point during the resource lifecycle. In this case, the *collection purpose* of one or different parent resources can record aggregation conditions, which can specify whether a particular agent is allowed to perform an aggregation (activity) on any given resource or a set of resources (entities), or if there are limitations for particular agents, entities, or activities regarding certain transformations. However, often resources in large-scale infrastructures may not have explicitly defined *collection purposes* for every transformation, so they can inherit *collection purposes* from their parent resources if allowed. Therefore, we suggest deriving an *implicit collection purpose* from the provenance of the parent resources. The *implicit collection purpose* of the aggregated entityABC is a UNION set of the *collection purposes* of all the parent entities A, B, and C. Table 1 shows the valid *collection purposes* of entities A, B, and C. However, in order for an agent TLE to access the entityABC, its *access purpose* must be a subset of the intersection set of all the parent entities A, B, and C. This way the aggregated resource may have a larger set of *implicit collection purposes*, however for a DP or an agent to access the aggregated resource, it must have an explicit *access purpose* that is either predefined or a common set of all parent entities, which in this case is “traffic law enforcement”.

It is important to note here that generating an *implicit collection purpose* is only suitable if the system has pre-defined rules for managing personal data. For instance, a new resource whose parent resources have the same DC or are collected under similar legal bases, or a resource with the same set of DS, can have an aggregation of *collection purposes* and thus an implicit *collection purpose* can be derived. An *implicit collection purpose* should only be derived if the explicit aggregation conditions are either not mentioned or parent resources have allowed the derivation of an implicit *collection purpose*. For example, if one of the parent’s *collection purpose* is supported by legal base public interest, while the other parent’s *collection purpose* is supported by informed consent, then the latter DC of the aggregated entity must acquire the consent of the DS (for the aggregation) in order to make the resource accessible to the DP, if not explicitly stated otherwise. If the parent entities’ *collection purposes* are supported by public interest or legal obligation, then the DC does not require explicit consent from the data subjects (DS), if it has the authorization to access parent resources (one or many) for the given *collection purpose/s*.

Once the transformed or aggregated resource/entity has a designated *collection purpose* (either implicit or explicit), the next step is to verify it against the *access purpose* of the agent or DP to check if access to the resource can be allowed for the given *collection purpose*. In order to achieve this, an access control module (ACM) of the system needs to consider both the *collection purpose* of the resource (entity) and the *access purpose* of the DP (agent) while making an access control decision. This will be discussed in the next section.

## 4. Provenance-Enabled Access Control

An access control module (ACM) regulates the access to system resources in order to control the flow of information to different system users or DPs. An ACM typically has four components: users, resources, reference modules, and access control policies. A reference monitor evaluates the properties of both users and resources against the access control policies to make a decision. ACMs are broadly categorized into two types: role-based access control (RBAC) and attribute-based access control (ABAC) [14,15]. In traditional RBAC, users with a similar set of properties (name, ID, location, role-type, etc.) will be given the same level of access to requested resources. For example, any user with a property “role-type” as “traffic-monitoring DP” will be allowed to access a resource (video recordings) from the cameras installed at a location, for example “north-east highway”. In traditional ABAC, the properties/attributes of all users, resources, and the system (environment) are taken into account while making an access decision; moreover, the decision to access a resource may vary according to the current requirements of the user, resource, or system at any given time [15]. For instance, along with the location, “north-east highway”, users can be further restricted to access a resource (video recordings), only recorded within their “duty hours”. ABAC solutions are more flexible than RBAC solutions, though the policy mechanism is complex if the dynamic properties and attributes are greater both in number and dimensions (belonging to multiple users and resources), thus making it hard for adoption in large-scale distributed systems. However, on the positive side, ABAC can accommodate many different types of resource properties (metadata, provenance, *collection purpose*, etc.), that can be defined in (explicit) permissions for a resource policy, thus, it can play an important role in decision-making. On the other hand, RBAC solutions have a fairly easy access policy mechanism, making them a feasible choice for any infrastructure with a defined set of users. However, only using RBAC will not accommodate the varying factors present in large-scale infrastructures where resources are transformed and aggregated multiple times, potentially changing their nature and attributes, thus requiring a change in RBAC access policies defined for users every time. To cater to the concerns of using RBAC and ABAC exclusively, several ACM solutions use both RBAC and ABAC in a logical combination to serve the needs of the system, such as an identity-based access capability (ICAP), trust-based access control (TBAC), provenance-based access control (PBAC) [16,17], etc. It is important to distinguish here between PBAC and provenance access control (PAC). The former uses provenance data to make an access decision, while the latter deals with regulating access to provenance as a resource PAC [18,19]. Moreover, in PBAC, provenance has a limited capacity to store and represent different properties of both the user (agent) and the resource (entity), therefore, in large-scale infrastructures, it is unlikely that PBAC can be used as the only ACM, and it is often coupled with RBAC or ABAC. Therefore, keeping in view our requirements for a large-scale infrastructure ACM that also prevents secondary use, the ACM needs to consider different resource properties (provenance, *collection purposes*, etc.) in case of transformations and aggregations along with an accommodating an access policy mechanism for users/DPs with emerging requirements, while making a decision.

One such RBAC–ABAC hybrid solution that is designed according to large-scale infrastructure requirements is the attributes enhanced role-based access control (AERBAC) model, as presented in the initial paper [20]. It uses RBAC for its dynamic role-assigning simplicity in categorizing users (DPs) and assigning them minimum default permissions as per their role and then utilizes ABAC for evaluating different resource and system properties thus implementing fine-grained access. AERBAC also uses *collection purpose* as a resource property and as part of its decision-making in another adaption of its model. However, in its current form, it cannot be ensured that the presented *collection purpose* is as agreed-upon and is not transformed or misinterpreted, thus disfavoring the purpose of compliance. Therefore, in order to ensure that the *collection purpose* is not lost or misinterpreted, we propose the use of provenance as a resource property and further extract the *collection purpose* from this to be used in the decision-making process. This provides two benefits: first, the integrity of *collection purpose* is preserved as it a part of immutable provenance metadata, thus ensuring that if it is compared with the *access purpose* then the resource (or the personal information present in resource) is only used for the agreed-upon *collection purpose* and secondary use is discouraged. Secondly, a DC can always track resource provenance to review how the resource has been used and for which collection and *access purposes*, using this information to assure DSs about their data usage and thus increasing their trust in their respective DC.

**Figure 4:**
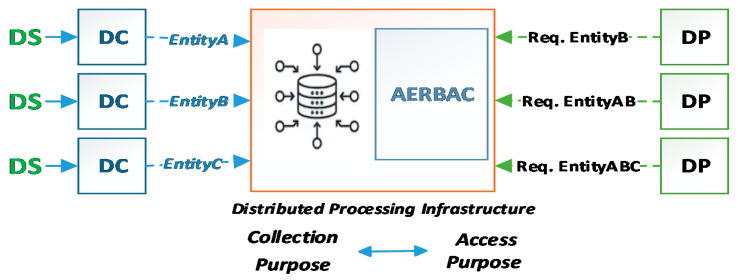
Provenance-enabled access control model.

To describe how this is achieved, a brief scenario is discussed below, as shown in Figure 4. The DC collects data from the DS with a certain *collection purpose* and then processes that data (resource) as per its requirements and transforms it accordingly. The resource (transformed data) along with its provenance is shared with the distributed infrastructure, which aggregates resources from many different DCs. A resource may go through different transformations and aggregations, and the provenance of the resource will be appended accordingly. As proposed above, if the provenance records the *collection purpose* as a property, and a resource goes through a transformation that does not affect data properties, the *collection purpose* will be left unmodified. However, if the resource is aggregated with some other resource that may change the nature or affects its data properties, then their provenances are stitched together, thus combining and appending the *collection purpose* for the newly aggregated resource [21,22]. Here, the ACM (AERBAC) can verify an authorized DP, allowing them to request a certain resource or not. Furthermore, it will ensure that the resource is not being used for any *access purposes* other than the one that is a subset of resources’ *collection purpose* recorded in its provenance. Moreover, provenance metadata is also considered sensitive, thus it only should be accessible to an authorized DP, preserving PAC. For instance, as shown in Figure 4, resources (entityA, entityB, and entityC) are managed by different DCs and are introduced to or shared with the distributed infrastructure along with their provenance. The DP is an authorized user and requests the shared infrastructure for an aggregated resource (entityABC) according to the *access purpose.* EntityABC inherits provenance from all its parent entities and now has a set of all their *collection purposes*. In order to ensure purpose limitation on the data (personal information), the DP’s *access purpose* should either be a subset of the resources’ *collection purpose* or part of all the parent resources of the newly aggregated resources’ *collection purposes*, as discussed in Section 3.2.

**Figure 5:**
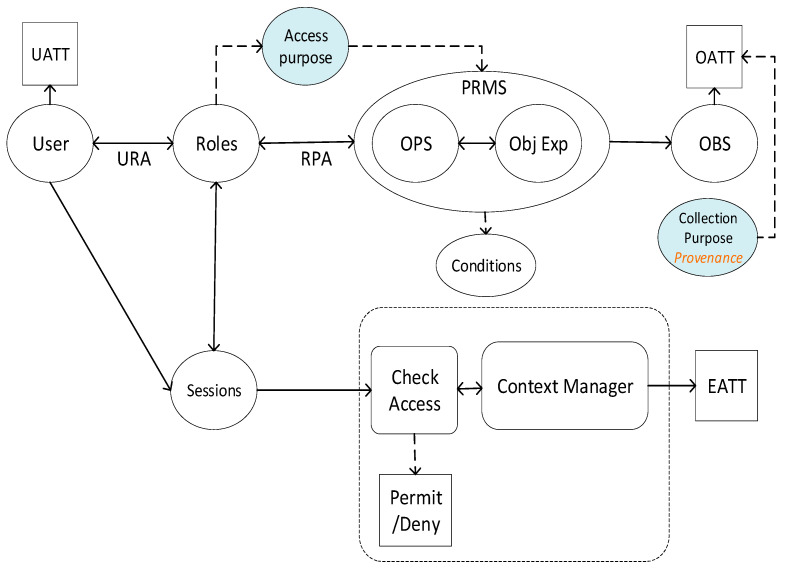
Provenance-enabled AERBAC.

Here, we will realize the above-stated model with our previously proposed AERBAC, an ACM designed for large-scale distributed infrastructures with added modification of using the *collection purpose* retrieved from provenance as a resource property, as shown in Figure 5.

### 4.1. Provenance-Enabled AERBAC

Users/DPs have various user attributes (UATTs) that describe them to the system. One of these UATTs is their role, which describes their authorized requirements. A DP can assume multiple roles based on their requirements or changing contexts, and AERBAC with dynamic role-assignment supports this, i.e., a finite number of roles are defined and any DP, based on relevant conditions and requirements, can be assigned an applicable role. This helps in limiting the total number of roles based on dynamic or contextual conditions as compared to declaring various roles exclusively for all DPs any time their requirements are modified. For every role, there is a set of permissions (PRMS) defined in terms of resource and system (environmental) attributes outlining conditions that need to be fulfilled in order to gain access. PRMS also includes an *access purpose* that is required if the requested resource contains personal information. This part of AERBAC is implemented as an ABAC, as permissions refer to resources indirectly, i.e., they are not bound to specific resources rather they define a resource in terms of attributes. Moreover, a system may also have a large number of resources that have been contributed by different DCs, referred to as an OBS, and have different attributes (OATTs) identifying themselves to the system. Some UATT and OATT attributes are dependent upon the system environment or surroundings and are called environmental attributes or EATT. OATTs often label different data properties describing types of information present in the OBS and there can be other OATTs that describe characteristics such as *collection purposes* (retrieved from provenance). All these different attributes (UATTs, OATTs, and EATTs) are used in different permissions and conditions assigned to different OBSs and roles that need to be fulfilled if an OBS needs to be accessed. These attributes are described in detail in the original papers that introduced AERBAC [20]. For every OBS, a DC constructs a set of object expressions (Obj. Exp.) that formally describe the requirements that need to be fulfilled in order for an OBS to access specific operation (OPS), i.e., insert, edit, append, etc. Obj. Exp. bound with an OPS create permission (PRMS) for a specific role against a particular OBS. Every PRMS consists of one or more conditions that need to be satisfied by the DP/user when requesting an OBS. At the time of the request, a unique session is created for the user against their assigned role. Once it is established that the user is authorized to access the requested OBS, the system retrieves the provenance (or the latest node of the provenance data) of the OBS that contains the current or latest append-only *collection purpose*. The system then compares the user’s *access purpose* with the OBS’s *collection purpose* to determine the information exposure for the particular user.

Thus, AERBAC utilizing RBAC for defining responsibilities and *access purposes* for users with diverse requirements, and ABAC for using OBS properties including *collection purpose* in access decisions, can help verify them against each other at a fine-tuned level, ensuring purpose limitation. Furthermore, provenance-enabled AERBAC adds another privacy layer by preserving the purpose integrity as an OATT, which validates resource or OBS usage transparency and shows compliance with data protection legislation guidelines. In addition, when *collection purpose* as an OATT is preserved in provenance through different transformations and is readily available when required in its agreed-upon state, purpose integrity is ensured. Moreover, implicit *collection purpose* can be derived in case of aggregation, eliminating the explicit and continuous creation of new *access purposes* for roles and providing flexibility for a DP with evolving requirements to access transformed or aggregated resources and without constant authorization. This makes AEERBAC an apt choice for large-scale and distributed infrastructure dynamics, as DPs/roles are not bound to resources but to *access purposes*, giving the flexibility of adding, removing, or transforming DPs at any point in the system without changing permissions against any role [23].

### 4.2. Analysis and Discussion

We will analyze the above-presented provenance-enabled AERBAC model by applying it to the motivation example discussed in Section 2. Let us say that an SC-TMS has various OBSs, such as video-recordings (entityA), vehicle GPS readings (entityB), vehicle registration data (entityC), etc., with different (OATTs) and their common *collection purposes*, as discussed in Table 3 and Table 1, respectively. EntityA is a video surveillance recording: a recording at a certain location for a specific time interval which does not have defined data characteristics. Yet, based on the processing capability of extracting different types of information, OATT can be identified as object-type (human, vehicles), object-descriptive features (gender, color, estimated age, and height), time-of-recording, compression-ratio, camera-type, camera-elevation, etc. EntityB represents vehicle GPS readings that record vehicle identification ID with its relevant reading. The OATT of entityC, which is a structured database of vehicle registration data, records license and registered vehicles for individuals or DSs.

The aggregated entityABC thus inherits OATTs from all its parent entities and has a large set of attributes that can be categorized or used in different combinations to extract useful information. For instance, vehicle ID from entityB can be matched with the vehicle ID of entityC to ascertain the vehicle owner or location of a vehicle at any given time. Similarly, if an event is recorded in an entity such as “speeding”, then the vehicle captured in this recording can be matched with the vehicle ID in entityC to register a violation. Thus, the aggregated entityABC offers a lot of personal information about individuals/DSs in different ways. However, the personal information should only be used for its *collection purpose,* which again entityABC inherits from its parents. As we proposed earlier, if explicit *collection purposes* are not defined for entityABC and there is a common *collection purpose* among all the parent entities, it can be used as an implicit *collection purpose*, which in this case is “traffic law enforcement”, as detailed in Table 4.

In our previous paper, we defined the basic characteristics for defining *collection purpose* as a provenance property. The first characteristic of a *collection purpose* declares the data attributes/properties of the entity that holds personal information [10]. Secondly, a set of properties are mapped to a specific purpose or function, describing input and output properties. Thirdly, for every function, a set of compliance policies are defined that need to be followed if the properties bound to that function are to be accessed [24]. A resource can have one or multiple functions for a *collection purpose*. Lastly, it records aggregation limitation, if there are explicit conditions to be followed in the case of resource transformation or aggregation. It also stores the legal frameworks supporting the *collection purpose*. All these properties are described in Table 4. The second column of the table shows some examples of object expressions that can be used for requesting specific resources or entities from parent resources/entities, which will also be valid for the aggregated entity entityABC, used for the implicit *collection purpose*. Thus, AERBAC allows all the relevant object expressions (Obj. Exp), conditions, and permissions (PRMS) that were designed for parent entities to be used for the aggregated entity, if requested for the implicit *collection purpose*.

A DP with an authorized role “traffic law enforcement system” has a UATT *access purpose* that allows it to access entityA (video surveillance recording) and entityC (vehicle registration data) exclusively, as shown in Table 5. Therefore, it can request the aggregated entityABC for an *access purpose* such as “issue fine for a traffic violation”, as this is allowed in the implicit *collection purpose* of entityABC, if not defined otherwise. On the other hand, the same DP also has an *access purpose* relevant to the *collection purpose* of entityC that is “registration of a new or unregistered vehicle”. However, the DP cannot use the data from entityA, i.e., a recording showing an unregistered vehicle and registering that vehicle in entityC, as this is not allowed in the *collection purpose* of entityA. Thus, AERBAC, by comparing the *collection purpose* of the aggregated resource with the *access purpose* of the agent, allows implicit access and ensures purpose limitation by preventing secondary use [25].

**Figure 6:**
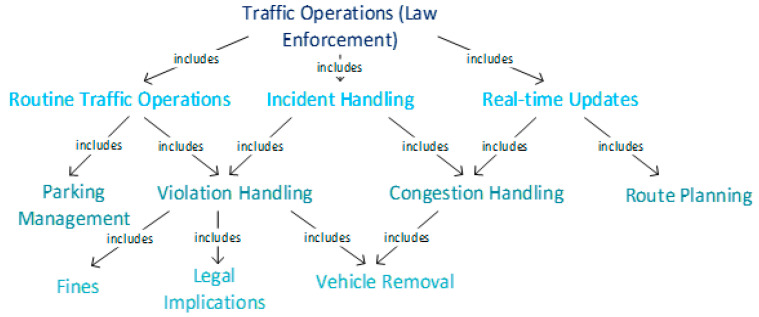
Implicit *collection purpose* hierarchy for an SC-TMS.

It is possible that a common *collection purpose* is not available, as in the above-mentioned example; however, often in the case of aggregations or transformations, there is some cohesion or similarity that acts as a motivating factor for the combination of the data for the enrichment of the existing information [26]. For instance, in an SC-TMS, most of the *collection purposes* are regarding traffic operations and management and are collected under the same legal base of public interest. In such cases, a basic implicit *collection purpose* hierarchy can be created for the aggregated entity, as shown in Figure 6. *Collection purposes* can be arranged in order of the highest number of OATTs describing personal information to the lowest number of OATTs with personal information. Thus, even if the entities do not have a common *collection purpose* at the same level of the hierarchy, it is possible that an authorized DP with a valid *access purpose* will be allowed to access the aggregated resource for a purpose lower in the hierarchy. It will limit the exposure of information to the DP and yet allow them to access the OBS without redefining permissions for this DP. Hence, provenance-adapted AERBAC allows a DP with existing an *access purpose* to use transformed or aggregated resources without violating any *collection purposes*. For instance, if an aggregated entity has a set of *collection purposes* containing “routine-traffic operations”, “incident handling”, and “real-time updates”, then a DP with an *access purpose* similar to “violation handling” can request the aggregated entity for access to certain OATTs. As a result, the DP will only be allowed to access information relevant to their *access purpose*, i.e., “violation handling” as a subset of the shared *collection purpose* of some of the parent entities, allowing limited disclosure only about that specific information. Thus, DPs are allowed access to new or aggregated resources with existing permissions limited to their *access purposes*.

To summarize, AERBAC with its dynamic role assignment and ABAC-based resources permissions is a suitable choice for implementing an efficient ACM in large-scale infrastructures [23]. Moreover, provenance-enabled AERBAC ensures purpose limitation by using *collection purposes* with preserved integrity as part of its access decision mechanism. After a DP is authorized to access a resource, AERBAC then verifies the *collection purpose* of the requested resource against the *access purpose* of the DP to ensure that the resource is being used as agreed. In the case of aggregated resources, where an explicit *collection purpose* is not defined, an implicit *collection purpose* can be derived from the common set of *collection purposes* of all the parent resources to allow authorized access to the DP.

## 5. Related Work

In this paper, our proposed approach emphasizes two key ideas: first, that provenance can be used to store the *collection purpose* of resources that contain personal information, second, that provenance-recorded *collection purposes* can be used in large-scale access control mechanisms to prevent secondary use and ensure compliance. Therefore, in this section, we discuss various state-of-the-art solutions that use provenance in some form as part of their access control mechanism. Furthermore, we will also briefly discuss different methods that are used for purpose limitation in large-scale infrastructures.

In the last decade, a staggering number of applications and services based on distributed infrastructures and cloud technologies have highlighted the importance of provenance. Over the years, different provenance schemes have been proposed to describe a way to show data lineage and derivation [18,21]. These schemes may offer a different view of provenance metadata based on its use, i.e., debugging, reproducibility, annotation, security, etc., thus, provenance along with data lineage information may store other characteristics as required for the usage purpose [27]. Provenance has also been used in different enhanced access control approaches for different storage and distributed platforms [28,29,30,31]. Some ACM solutions propose to capture resource provenance during different activities and then use this information in access control solutions: this is generally referred to as provenance-based access control (PBAC) [30]. One such notable contribution extracts resource dependencies from provenance logs and uses them to authorize and authenticate users in distributed cloud environments, and later uses this as an attribute in ABAC to make access decisions [31]. In another approach, the authors proposed a generic ontology to capture semantic information (attributes) from different provenance schemes present in distributed infrastructures, subsequently basing classified resources on this information in order to assign access privileges to classified resources [32]. Provenance can also help in the implementation of organizational security policies and is proposed as a hybrid approach with ABAC for enforcement [28,29,30,31]. An architecture for cloud infrastructure has also been developed that utilizes contextual information derived from provenance metadata for evaluating policy decisions [33]. Thus, provenance has been used in different ways for enabling ACM with either deriving policies from resource dependencies or authorizing users, but to our knowledge, it has not been used in ACMs to control personal information usage in resources, i.e., in purpose limitation to restrict secondary use, as we have proposed.

Purpose limitation in nutshell is the limitation of users/DPs in accordance with DS preferences, and there are different ways of achieving this as proposed in the literature [34,35,36,37,38]. The most common approach is to define compatible purposes for both resources (collection purposes) and users (*access purposes*) to maintain a hierarchy with certain privileges bound to different purposes in the system [35]. Whenever a user requests a resource for a given purpose, its purpose is compared to the purposes in the system hierarchy, and if matched, then those privileges are authorized for that user. Different solutions customize those privileges to ensure the fine-tuning of access to resources, for instance, in a relational database where data properties are distinctively labeled into different properties and purposes are bound to them [38]. In another approach, purposes are divided into three classes: allowed purpose, conditional purpose, and prohibited purpose, which are assigned based on changing contextual attributes in dynamic role assignment. However, many of these solutions require predefined knowledge about resources and users and a DC to directly authorize users certain access privileges for the purposes associated with a resource. Our proposed approach decreases the reliance on DCs for this authorization of users/DPs and supports transformational changes in resources that may affect the *collection purposes* of the resources.

## 6. Conclusions

Provenance catalogs information about different activities performed by a resource, since its generation and the part of it that contains information about the resource lineage is usually immutable. In this paper, we proposed the use of provenance for purpose limitation within distributed infrastructure that processes personal information in some form by making the *collection purpose* a part of immutable provenance. As provenance is a part of a resource, and *collection purpose* is a part of provenance, therefore, whenever the resource is transformed or aggregated with another resource its *collection purpose* is inherited and preserved. When the resource is requested for access, its *collection purpose* can be compared with the *access purpose* of the user or DP, thus helping to verify that the resource is being used for purposes it was collected for, thus ensuring purpose limitation.

## Figures and Tables

**Table 1:** Data *collection purposes* and legal base.

Data Source (with Personal Information)	Legal Base	Likely Collection Purposes for Aggregated Resource
Video Surveillance Data (Unstructured)	Public Interest	Public safety Traffic management Real-time traffic updates Route planning Traffic law enforcement
Vehicles’ Sensor Data from Public Transportation Modes (Semi-Structured)	Contract	Vehicle tracking Congestion handling Weather monitoring Noise reduction Real-time traffic updates Route planning Traffic law enforcement
Vehicle registration data (structured)	Legal Obligation	Vehicle registration License registration Incident handling Violation handling Traffic law enforcement

**Table 2:** GDPR articles supporting purpose limitation.

GDPR Article Info.	Description
Data minimization (9, Article 5, x1(c))	“(Personal data shall be) adequate, relevant, and limited to what is necessary for relation to the purposes for which they are processed (...)”
Purpose limitation (9, Article 5, x1(b))	“(Personal data shall be) collected for specified, explicit, and legitimate purposes and not further processed in a manner that is incompatible with those purposes (...)”
Access control (9, Article 25, x1)	“The controller shall implement appropriate technical and organizational measures for ensuring that, by default, only personal data, which are necessary for each specific purpose of the processing, are processed (...) personal data are not made accessible without the individual’s intervention to an indefinite number of natural persons.”
Legal Base	“Consent should be given by a clear affirmative act establishing a freely given, specific, informed, and unambiguous indication of the data subject’s agreement to the processing of personal data relating to him or her (...)”
Consent (9, Recital (32))	“(...) the controller shall have the obligation to erase personal data without undue delay where one of the following grounds applies: the personal data are no longer necessary in relation to the purposes for which they were collected or otherwise.”
Right to be forgotten (9, Article 17 x1)	

**Table 3:** OATT and EATT of an SC-TMS.

OBS	OATT	EATT
Video Surveillance Recording (entityA)	Time-of-recording, compression-ratio, camera-type, camera-elevation, events-type-detected-in-recording, objects-type-detected-in-recording, object-type (human, vehicles), object-descriptive-features (gender, color, estimated age, and height), object-identification-features (face, gait, license plate), geo-location data (spatiotemporal position of any object at a specific time), camera-locations (highways and others along the road capturing traffic only), devices (video camera types and unique IDs), timestamp-of-the-recording, etc.	Recording Location Time-of-recording
Vehicle GPS Readings (entityB)	Vehicle-ID, GPS-reading, geo-location data (spatiotemporal position of a vehicle at a specific time), timestamp-of-the-recording, etc.	Reading Timestamp Reading Location
Vehicle Registration Data (entityC)	Vehicle-ID, owner-ID, registered-license-plate, owners-driving-license-ID, violation-history, etc.	Vehicle Location

**Table 4:** An implicit *collection purpose* for OBS (entityABC).

Implicit Collection Purpose for OBS (entityABC) Traffic Law Enforcement	Obj. Expression (Examples)
*As entityABC is an aggregation of three OBS so it inherits (OATT) from all three parent entities. Only OATT that can store personal information is mentioned in ‘*collection purpose*’. **1.Personal Data Properties:** (entityA) OATT: Object-type (human, vehicles), object-descriptive-features (gender, color, estimated-age and height), object- identification-features (face, gait, license-plate) (entityB) OATT: Vehicle-ID, geo-location data (Spatio-temporal position of a vehicle) (entityC) OATT: Vehicle-ID, Owner-ID, registered-license-plate, Owners-driving-license-ID **2.Personal data property to function Mapping:** (Vehicle’s License plate, driver’s face) -> (are bound to functions {traffic light violation, Speeding vehicle, Wrong parking, Wrong turn, Driving in a bus lane, Junction-box violation}) (Vehicle’s License plate, driver’s and passengers’ face)-> {Accident/ Vehicle collision, Seat belt, child detected without a child seat, etc.} *An exhaustive list is defined for different agreed-upon functions and are bind to the required OATT describing personal information **3.Compliance Policy:** will be used for public interest reasons: ▪ To record, process, and store (activities) any event or object that demonstrates a Traffic operations or violation (traffic light violation, Speeding vehicle, Wrong parking, Wrong turn, Driving in a bus lane, Junction-box violation, Accident/ Vehicle collision, Seat belt, child detected without a child seat, etc.) ▪ To record, process, and store any event or object that demonstrates passenger handling, incompliance to traffic regulations, hinders/stops the routine or smooth traffic operations (function/sub purpose) ▪ To record, process, and store events and object involved in routine traffic operations function/sub purpose) ▪ To record, process, and store events and object involved in parking management function/sub purpose) **Cannot be used for tracking any event or object that is not mentioned in ‘purpose’ unless otherwise authorized by another legal base or higher authorized DC **4. Aggregation Limitation:** The said resource when aggregated with any other resource requires specific authorization from public-authority DC supported by a legal base of Consent, Legal obligation, or Vital Interest if used for the following functions/sub purposes. ▪ Link a license plate (OATT) to a unique DS ID, name, face (OATT) ▪ Link the descriptive features (OATT) of a human to the identification features (OATT) of a unique DS, ▪ Link the descriptive- features (OATT) of a human to the geo-location features (OATT) of a unique DS, **5.Legal Base**: Public Interest	Example 1 **Description**: Video-recordings that contains event-type “Speeding” (OATT) at location (EATT) “east highway” at the current time (EATT) **Formal**: Loc-type (EATT) = “East Highway”) ^ (Event (OATT) INCLUDES “speeding”) ^ (timestamp (EATT) current. Timestamp) Example 2 **Description**: Video-recordings that contains event-type “trespassing” (OATT) at a location (EATT) “town-museum” from December 1, 2020- December 15, 2020 (EATT) **Formal**: Loc-type (EATT) = “East Highway”) ^ (Event (OATT) INCLUDES “speeding”) ^ (timestamp (EATT) ” ^ (timestamp(o) AFTER 2020.12.01 00:00:00 BEFORE 2020.12.15 00:00:00) Example 3 **Description**: Insert fine for licensed-owner of the vehicle with event-type “Speeding” (OATT) **Formal:** OBS event-type “Speeding” (OATT) ^ object-identification = “license-plate”-> Operation (OPS) INSERT fine (OATT) FOR “license-plate-> licensed owner” = “licensed owner”

**Table 5:** *Access purpose* for DP/user (traffic law enforcement system).

Role UATT	Access Purpose (Issue Fine for a Traffic Violation)	Authorized OBS	Conditions
Traffic-Law Enforcement System	1. Detect and identify traffic event that is considered a violation either via video recording or senor-reading (e.g., speeding) 2. Identify the object-type vehicle (through its license plate or driver’s identification information) from video data, and in case of a detected traffic violation issue a fine and penalty points to the object-type driver, if applicable.	Video Surveillance Recordings, Vehicle Registration Database, Real-Time Updates From Traffic-Related Sensors	Licensed-owner (OATT) of entityC Vehicle Registration is equal to the license plate (OATT) of entityA value of object-type associated to the “Event-type” = traffic violation AND [“Event-type” (OATT) of the OBS video-recording is a traffic violation OR “Event-type” (OATT) of the OBS traffic sensors is a traffic violation]

## Data Availability

Not applicable.
